# MALIGNANT CATATONIA IN MEDICAL WARDS: A BROAD DIFFERENTIAL DIAGNOSIS

**DOI:** 10.1192/j.eurpsy.2023.1626

**Published:** 2023-07-19

**Authors:** N. Arbelo

**Affiliations:** 1 Psychiatry, Hospital Clinic of Barcelona, Barcelona, Spain

## Abstract

**Introduction:**

Catatonia is an uncommon and heterogeneous psychomotor syndrome. It can be not only the manifestation of a psychiatric disorder but also a wide range of medical conditions. The malignant catatonia is a subtype of catatonia which includes dysautonomic signs such as hyperthermia or hemodynamic instability, and because most of the affected patients are taking antipsychotics or antidepressants previously, it can be confounded with medical conditions such as neuroleptic or serotonin syndrome.

**Objectives:**

To present a case of malignant catatonia admitted in a medical ward

**Methods:**

The present study is a case report of a patient admitted with initial diagnosis of serotonin syndrome in a medical ward of our hospital and referred to the consultation and liaison psychiatry (CLP) unit. We also searched previously case reports, series and systematic reviews about catatonia secondary to medical conditions and hyperthermia catatonia.

**Results:**

Ms. TN is a 71-year-old woman, with prior history of major depressive disorder. One month ago she was admitted in a psychiatric ward of another hospital for a depressive episode with psychotics features, and was treated with escitalopram 10mg/day, vortioxetine 10mg/day, mirtazapine 15mg/day, trazodone 50mg/day, quetiapine 700mg/day and haloperidol 5mg/day. She had a worsening of depressive symptoms with suicidal thoughts, negativism and psychomotor retardation, and subsequently hyperthermia, rigidity, mydriasis, tachicardia and increased bowel sound. She was tranfered to our medical ward, and diagnosed of serotonin syndrome. She was stopped all the psychiatric drugs and was treated with dantrolene and support measures. After 10 days without antidepressants or antipsychotics she maintened the same symptomatology and was referred to our CLP unit. The psychopathological evaluation showed stupor, mutism, waxy flexibility and negativism, and she responded to a challenge test with intravenous clonazepam 0,5mg. She was diagnosed of malignant catatonia and was started oral clonazepam 2mg/day. Although there was a partial response, she did not tolerate higher doses because of sedation and finally was treated with electroconvulsive therapy (ECT). She dad a remission of catatonic symptoms after only two sessions of ECT.

**Conclusions:**

Malignant catatonia can be confounded with other medical conditions such as serotonin or neuroleptic syndromes. All of them can have catatonic signs, and it is important to recognize them (a challenge test with a benzodiacepine can be helpful). The key to distingish malignant catatonia from them is that some of the catatonic signs (negativism and psychomotor retardation) happened before the dysautonomic signs. Also, it is uncommon that a serotonin syndrome persisted more than 3-5 days after the suspension of antidepressants. Consultation and liaison psychiatrists can help for the differential diagnosis and management of patients with suspected catatonia in medical wards.

**Disclosure of Interest:**

None Declared

